# O063. Moyamoya disease and headache: case report

**DOI:** 10.1186/1129-2377-16-S1-A73

**Published:** 2015-09-28

**Authors:** Elisabetta Tozzi, Annarita Antenucci, Simona Di Loreto, Maria Maresca, Giovanni Farello, Luca Massimi

**Affiliations:** Departments of Life, Health and Environmental Sciences, Neuropsychiatric, University Hospital of L'Aquila, L'Aquila, Italy; Departments of Life, Health and Environmental Sciences, Pediatric, University Hospital of L'Aquila, L'Aquila, Italy; Agostino Gemelli, Policlinic, Rome, Italy

## Background

Moyamoya disease is a vascular dysplasia clinically characterized by a combination of ischemic and hemorrhagic strokes. The Research Committee on Moyamoya disease in 1979 classified the initial attacks into 6 types; subsequently, the asymptomatic and the headache type were also added in 2003. In fact headache is common in Moyamoya disease and can be the first symptom especially in childhood; its characteristics and classification are largely unknown because of the uncertainty and probably multifactorial nature of the mechanism[1].

## Case report

A seven-year-old Romanian girl, presented to the Emergency Department of the L'Aquila Hospital suffering from drop attacks, dysarthria, choreic movements and headache. Neurologic symptoms had appeared for four days and headache onset had started a year ago, with daily onset, especially in the evening, more pronounced in the frontal region, with oppressive and binding features, often triggered by physical exertion and requiring daily assumption of ketoprofene. The brain magnetic resonance imaging showed: bilateral cortical ischemic areas involving the fronto-mesial regions and the right perirolandic region, complete occlusion of the medium and posterior cerebral arteries, and lenticulostriate and thalamostriate arteries dilated with a “puff of smoke” appearance. The patient was sent to the Pediatric Neurosurgery of the A. Gemelli Hospital in Rome where she underwent cerebral arteriography and surgical operation. Indirect revascularization of the right (December 2014) and the left side (February 2015) was done by means of encephalo-myo-synangiosis and accessory burr holes. At first follow-up: the drop attacks had disappeared, and there was significant improvement of the hemiparesis and the steppage. Current therapy is cardioaspirin 50 mg/die. The girl refers considerable improvement of the headache with weekly recurrence and mild intensity.

## Discussion

Diagnostic classification of headache associated with Moyamoya disease is controversial: the characteristics of headache vary and may be migraine-like throbbing pain or the dull headache noted in tension-type headache. In most of the studies headache is suggested to be an important symptom of ischemic cerebrovascular diseases and surgery bypass seems to be an effective therapy in most patients. Other studies report that headache can persist or develop after indirect bypass surgery despite successful prevention of cerebral ischemia; thus, progressive recruitment and redistribution of blood flow should be considered another cause of headache. The efficacy of surgery revascularization, even on the headache symptom, clarified that her headache was probably related to cerebral hypoperfusion.

Written informed consent to publication was obtained from the patient(s).
